# Rural labor migration and household waste separation willingness: Evidence from 6849 rural residents in China

**DOI:** 10.1371/journal.pone.0321459

**Published:** 2025-05-28

**Authors:** Yi Yu, Dan Pan

**Affiliations:** School of Economics, Jiangxi University of Finance and Economics, Nanchang, China; Sichuan Agricultural University, CHINA

## Abstract

Understanding the key determinants of rural household waste separation willingness (HWSW) is indispensable in promoting sustainable development for many developing economies. Few studies have been dedicated to examining the impact of large-scale rural labor migration (RLM) on HWSW in rural China. Based on a nationwide sample including 6849 rural residents, this paper investigates the relationship between RLM and rural residents’ HWSW. The generalized propensity score (GPS) method and the instrumental variable (IV) approach are used to account for potential selection bias and endogenous problems. The results show that RLM inhibits rural residents' HWSW. Specifically, with every 1% increase in RLM, the likelihood of rural residents’ HWSW will decrease by 3.5%. This effect remains significant after a series of robustness checks. Heterogeneity analysis reveals that the negative impact of RLM on HWSW is larger for rural residents who live in Midwest China, with low education and less income. Further mechanism analysis shows that RLM reduces rural residents’ HWSW by decreasing their social capital, undermining their rural community administration, and diminishing their village's collective economic income.

## Introduction

Tremendous household waste generation has become an extremely dreadful problem worldwide, especially in developing countries with increasing economic growth, such as China and India [1,2]. It is estimated that the number of solid waste generated worldwide was only 2 billion tons in 2016 and is expected to achieve 3.4 billion tons by 2050 [3]. The excessive amounts of household waste have caused great detriment to the environment and people's welfare, which are a major threat to the accomplishment of the 2030 Sustainable Development Goals [4,5]. Compared with urban household waste management, rural household waste management is confronted with greater barriers because of the low population density, scattered waste distribution, troublesome transportation, and insufficient waste treatment infrastructure due to expensive costs and low service scale [6,7]. As one of the largest developing countries worldwide, with nearly 600 million rural residents, China has generated enormous amounts of rural household waste, which has become a major bottleneck for its sustainable development [8]. It is reported that household waste produced in rural China is about 180 million tons each year, with a harmless treatment rate of only 38.27% by the end of 2019 [9]. This issue is compounded by the economic rise in developing countries, which has significantly altered the consumption patterns of rural residents and led to a more complex composition of household waste [10]. For instance, increasingly urbanized lifestyles and consumption habits have driven a noticeable increase in disposable items, industrial products, and plastic goods [11]. At the same time, clothing and durable consumer goods, which were previously rarely discarded, are now a significant part of the waste stream. Similarly, leftover food, fruit peels, vegetable leaves, crop vines, and straws, which were once used as livestock feed, have now become waste. Together with the widespread use of plastics and industrial products, these changes have contributed to an increase in toxic, hazardous, and non-degradable materials in rural waste [12]. This is further exacerbated by the sheer scale of waste generation in rural areas. According to statistics, the permanent rural population in China exceeds 500 million. With an average daily waste generation of 0.8 kilograms per person, the total daily waste in rural areas amounts to 149 million tons, of which approximately 30% to 40% is not effectively treated [13]. Consequently, the impact of household waste on the rural ecological environment is becoming increasingly severe.

One way to overcome these problems is by promoting rural residents’ household waste separation behavior [14]. The implementation of rural residents’ household waste separation was initiated by the Chinese central government in 2016 and has been consistently emphasized in the No.1 Central Documents from 2020 to 2023. In 2022, multiple government ministries collaborated to issue a comprehensive policy requiring county-level governments to develop and manage rural waste systems effectively. This includes promoting waste separation and resource utilization at the source and improving related infrastructure. The central government has invested over CNY 25.8 billion (USD 3.6 billion) in rural waste disposal infrastructure [15]. However, the success of these measures largely depends on the active participation of rural residents, as they are the main executors and beneficiaries of waste separation [16]. Their involvement can alleviate high transportation costs [17], reduce land use [18], increase service efficiency [19], and improve environmental quality. Therefore, promoting rural residents' household waste separation willingness (HWSW) is crucial for the success of rural waste management.

Numerous studies have found that rural residents’ HWSW is affected by three factors. The first is socioeconomic factors, including gender [20], age [8], education [21], and income[22]. The second is psychological factors, such as waste treatment attitude [23], environmental protection awareness [24], knowledge [25], and subjective norms [26]. The third aspect is external contextual factors, including government policy [27] and infrastructure provision [19]. However, few published studies have been able to draw on any systematic research into the influence of rural labor migration (RLM) on HWSW.

In reality, RLM—rural residents migrating from rural areas to cities, is considered a remarkable characteristic in the development of rural China [28]. Driven by the urban-rural income gap and high economic benefits in cities, a large number of rural residents in China migrate to cities to pursue high incomes [29]. It is reported the total number of rural migration laborers was 286 million in 2020, accounting for 20.2% of the total population in China [9]. This massive RLM has greatly boosted rural economic development but also had profound impacts on rural environmental management in rural China [29]. For example, Zhang et al. [30] found that RLM can reduce fertilizer use in China. Xu et al. [31] also found that RLM has a positive impact on agricultural non-point source pollution reduction. Generally speaking, the above-mentioned literature mostly concentrates on the link between RLM and rural agricultural pollution management, not on rural household waste pollution management. In theory, RLM can transform rural residents’ livelihood strategy and their waste management awareness and thus may have an impact on rural household waste pollution management [29,32,33]. Therefore, carrying on quantitative analysis on the impact of RLM on HWSW is essential.

To fill the above gap, grounded on random survey data of 6849 rural residents in China, we conduct a rigorous quantitative estimation of the impact of RLM on rural residents’ HWSW and further test its underlying mechanism. Several models, including the Logit model, the generalized propensity score (GPS) method, the instrumental variable (IV) approach, and the mechanism effect model, are used to reach unbiased estimations. We discover that RLM has a significant negative impact on HWSW. After removing the selective bias, using the 2SLS model for the endogeneity test, changing the definition of HWSW and the regression method, and winsorizing extreme values, the conclusion is still robust. Heterogeneity analysis shows that the inhibiting impact of RLM on HWSW is larger for residents who live in Midwest China, with low-educated and low-income levels. Mechanism analysis shows that RLM reduces rural residents’ HWSW by lowering their social capital, undermining their rural community administration, and diminishing their village's collective economic income.

We make four contributions to literature. First, we provide a novel understanding on how migration reshapes rural residents’ pro-environmental actions, thus offering a new perspective on the intersection of rural migration studies and environmental governance. Existing studies on waste management have predominantly focused on urban areas, with limited attention to rural regions, where waste separation behaviors are shaped by unique socio-economic and cultural dynamics, and more in-depth research is needed. Moreover, prior research rarely considers the role of large-scale labor migration in influencing rural environmental behavior, leaving a critical gap in learning how migration is changing the rural residents’ pro-environmental actions. By providing a rigorous quantitative estimation of the impact of RLM on rural residents’ HWSW, our study bridges this gap.

Second, we analyze the underlying mechanisms through which RLM affects rural residents’ HWSW, both theoretically and empirically. We integrate the Theory of Planned Behavior (TPB) and community governance theories to explain how subjective norms (e.g., social capital and rural community administration) and perceived behavioral control (e.g., village's collective economic income) mediate the relationship between RLM and HWSW. This theoretical integration not only enriches the understanding of the behavioral mechanisms at play but also advances the literature by linking migration studies with environmental behavior research.

Third, we employ a large-scale and nationally representative datasets covering 6,849 rural residents across 27 provinces in China, thus offering insights that go beyond regional contexts. Compared to previous studies that often rely on small-scale or region-specific surveys, our datasets not only can provide a more comprehensive and accurate reflection of the real situation in China but also can represent the relationship between RLM and HWSW in regions. Thus, while our study focuses on China, our findings based on this large-scale datasets have broader implications for other countries undergoing modernization and urbanization, where rural decline and labor migration pose similar challenges to environmental governance.

Finally, we adopt rigorous methodological approaches to ensure the robustness of our results. Specifically, we employ the generalized propensity score (GPS) approach and instrumental variable (IV) methods to address potential endogeneity issues, such as sample self-selection bias, mutual causality, and un-observable factors. These advanced econometric techniques allow us to obtain unbiased and reliable estimates of the impact of RLM on HWSW, setting a methodological benchmark for future research in this field. Furthermore, the combination of GPS and IV methods demonstrates the feasibility of addressing complex endogeneity problems in studies of rural environmental behavior, providing a valuable reference for researchers exploring similar topics.

The rest of the paper is structurally arranged as follows. The theoretical analysis section proposes the theoretical hypotheses. The data, variables and model section presents the data, variables definitions, and model settings. The empirical results section presents the baseline results, the selection bias correction using the GPS method, the endogeneity analysis, robustness tests, heterogeneity analysis, and mechanism analysis. The final section exhibits the conclusions and corresponding policy recommendations.

## Literature review

Encouraging rural residents’ HWSW in China is challenging, and a large body of literature has been conducted to explore the driving factors. A summary of the literature on the determinants of rural residents’ HWSW in China is shown in Table 1.

**Table 1:** Literature review on the determinants of rural residents’ HWSW in China.

References	Statistical method used	Sample size	Main determinants of rural residents’ HWSW
**Ao et al.** [26]	Structural equation modeling	586 rural residents in 9 villages in Sichuan Province	Publicity and education, attitudes, subjective norms, past behavior, and sense of belonging
**Govindan et al.** [19]	Structural equation modeling	324 valid questionnaire responses in Shanghai	Attitude, subjective norms, perceived behavioral control, intention, and behavior
**Qing et al.** [20]	Binary logistic regression model	200 farmers in Sichuan Province	Age, gender, education, family size, household wealth
**Pan et al.** [27]	Poisson regression model and negative binomial model	150 samples from 2015 to 2019	Public-private partnership, fiscal transparency index, financial burden, financial development index
**Han et al.** [8]	Logistic regressions	811 households in 59 villages from six provinces in West China	Gender, demonstration, propaganda, treatment necessity, environmental pollution perception, age, education, income
**Liu and Wang** [22]	Logit-ISM model	671 surveys in Jiangsu	Income, sex, age, cadre status, attention to environmental protection information, identifying capacity for pollutants
**Meng et al.** [23]	Structural equation modeling	709 residents in Suzhou	Environmental attitudes, social norms, environmental knowledge, publicity and education, environmental facilities and services
**Liao et al.** [21]	Structural equation modeling	538 rural residents in the province of Sichuan in China	Age, gender, educational level, income

From Table 1, we can discover that there are three deficiencies regarding the determinants of rural residents’ HWSW. First, the existing literature mostly focused on how socioeconomic factors (such as gender, age, education, and income), psychological factors (such as attitude, awareness, knowledge, and subjective norms), and external contextual factors (such as government policy, and infrastructure provision), but the analysis of the impact of RLM on rural residents’ HWSW is limited. Second, most existing literature was based on the survey data of several provinces, lacking a systematic study at the national level. Third, most of the empirical studies employed the structural equation modeling, logit, or logistic model, which cannot deal with the potential endogenous problems between RLM and rural residents’ HWSW, and thus may generate biased results. To remedy the above deficiencies, this paper tries to complement these prior studies by investigating the impact of RLM on rural residents’ HWSW with a GPS approach and an IV method based on a nationwide sample including 6849 rural residents in 27 provinces in China.

## Theoretical analysis

### The impact of RLM on HWSW

This section explores how RLM affects HWSW. We analysis the RLM weakens rural residents’ HWSW by increasing waste management costs, reducing the participation capacity of left-behind groups, and decreasing migrant households’ attention to the rural environment. The detailed discussion is as follows:

First, RLM will increase the cost of rural waste management, thus impeding rural residents’ HWSW. This is because the service scale of rural waste management is minor due to the low population density and scattered waste distribution of rural wastes. Due to RLM, the population density in rural areas becomes even lower, and the cost of rural waste management becomes much higher [34].

Second, the large flow of RLM leaves older people, women, and children in rural areas [35]. These left-behind people are less educated [36] and relatively weak in environmental cognition [37]. They have a low acceptance of new knowledge, the latest technology, and fresh ideas, resulting in little enthusiasm to engage in rural environmental improvement, consequently reducing the possibilities of HWSW participation [38].

Third, rural families with a high degree of RLM spend less time living in villages, are less dependent on agricultural income, and are more accustomed to urban life. They will pay less attention to the rural environment, which will reduce the possibility of their participation in rural waste management [39].

Therefore, we put forward our **Hypothesis 1** that RLM will hinder HWSW.

### The mechanism between RLM and HWSW

The Theory of Planned Behavior (TPB) has been widely used in behavioral studies in different domains including the specific domain of HWSW [21]. TPB explains the general decision-making process of individual behavior based on the expected value and has been widely applied to interpret complex psychological and behavioral phenomena. Most studies confirm its effectiveness, especially subjective norms and perceived behavioral control, in enhancing the explanation and prediction of behavioral activities [40,41]. Therefore, this study focuses on analyzing the mechanism between RLM and HWSW through subjective norms and perceived behavioral control.

Subjective norms refer to the external social pressures an individual feels when deciding to perform a behavior [42]. In the context of rural waste separation behavior, rural residents’ subjective norms are influenced by pressures such as encouragement or opposition from local governments, recognition or discrimination by neighbors, and support or boycotts from rural organizations. Therefore, we use two indicators—social capital and rural community administration, as proxy variables to capture these subjective norms.

In terms of social capital, RLM weakens the social capital among rural residents, which in turn impedes their HWSW. Numerous pieces of literature verified that social capital plays a critical role in promoting rural residents’ HWSW [43]. For one thing, RLM reduces the frequency of interaction among rural residents, which results in weakened ties between outbound and in-village rural residents that lower HWSW. For another, the original relatively stable social capital mechanism among rural residents is also seriously damaged due to RLM, which results in lower HWSW [44]. Thus, the decline in social capital caused by RLM leads to a decrease in rural residents’ HWSW.

In terms of rural community administration, rural community plays a crucial role in rural waste management due to the self-governance system in rural China [45]. By assigning responsibilities to specific individuals or groups within the village and implementing supervision and punishment measures to increase the cost of non-compliance, rural community administration serves as a key governance model in rural communities and has an advantage in encouraging rural residents to adopt proper waste management practices [46]. However, RLM weakens rural community administration. This is because the outflow of young and capable rural residents weakens the enforcement of supervision and punishment and diminishes social incentives for compliance in the process of rural community administration. Consequently, the rural community administration capacity declines, leading to a reduction in rural residents’ HWSW [41].

Perceived behavioral control (PBC) is closely related to Bandura’s concept of self-efficacy, defined as “a judgment of one's capability to accomplish a certain level of performance” [47]. In the context of rural waste separation behavior, PBC reflects rural residents’ perceptions of factors that facilitate or hinder their participation, such as resource endowment, institutional support, and social resources [42]. The Chinese government rather than rural people is responsible for the construction and maintenance of rural waste management facilities and services [48]. Therefore, villages with higher village's collective economic income typically possess more financial resources to implement these public services and further enhance rural residents’ PBC by making rural residents’ participation in waste management more convenient and feasible [40]. However, RLM poses a significant challenge to the village's collective economic income. This is because the outflow of rural labor reduces the availability of human resources for the village's collective economic activities, leading to a decline in the village's collective economic income, which weakens the financial capacity of villages to sustain rural waste management systems, increasing the practical barriers perceived by residents. Consequently, the weakening of the village's collective economic income caused by RLM diminishes rural residents’ PBC, undermining their HWSW, despite the central government's efforts to provide external financial support.

Therefore, we put forward our **Hypothesis 2** that subjective norm (social capital, rural community administration) and perceived behavioral control (village's collective economic income) are possible mechanisms by which RLM negatively affects rural residents’ HWSW.

## Data, variables, and model

### Data

The data used in this study comes from the 2018 wave of the China Labor Force Dynamics Survey (CLDS2018) initiated by Sun Yat-sen University, which covers 29 provinces in China and establishes a three-level database of individuals, households, and villages. The respondents are the total labor force aged 15–64 in the sample households. Because the research object of our study is the household with RLM, after eliminating the non-labor migration families and missing variables, our benchmark sample is 6849.

### Variables

#### Dependent variable: rural residents’ HWSW.

The dependent variable is rural residents’ HWSW. In CLDS 2018, the respondents were asked: “whether your family willing to participate in waste classification?”. We use this for measuring HWSW and assign the value of unwilling to be 0 and willing to be 1.

#### Independent variable: RLM.

The independent variable is RLM. Referring to the study by Chen et al.[49], we denoted it as the rural labor force transfer rate, which is calculated as the number of people in the household minus the number of people engaged in agricultural production for more than three months, divided by the total number of people in the household. Because of the availability of relevant data in CLDS 2018, the labor force engaged in non-agricultural employment is defined as the total family labor force minus those engaged in agricultural production. This information is derived from the question in CLDS 2018: “Last year, what is the number of people engaged in agricultural production for more than three months in your family?”

#### Control variables.

Our control variables include three dimensions, which are individual characteristics, household characteristics, and village characteristics. Individual characteristics include gender, age, communist party members, marriage, and health. Household characteristics include household property, family members, and household sanitary conditions. Village characteristics include the villages’ social security conditions. All the corresponding questions come from CLDS 2018.

#### Mechanism variables.

Based on previous studies by Wang and Yang, and Zhu et al. [44,50], social capital is defined as the extent of a neighbor's familiarity, neighbor's trust, and mutual support, which can be measured from the following three questions in CLDS2018 “How familiar are you with your neighbors and other residents in this community, Do you trust your neighbors and other residents in this community, and Do you have mutual support with your neighbors and other residents in your village”. The answer to these questions both is “1=very little, 2=relatively little, 3=general, 4=relatively much, 5=very much”. Rural community administration is represented by whether the village adopts grid-based management, and the village's collective economic income is measured by the logarithm of the village's collective economic income.

#### Variables descriptive statistics.

Table 2 exhibits variable definitions and basic statistics. For the dependent and independent variables, we can find that over half of the rural residents are willing to engage in waste classification, with an average of 0.893. For the independent variable, the degree of rural labor migration (RLM) is moderate, with a mean value of 0.556. As for the control variables, the gender distribution is roughly balanced, with males accounting for 52.4% of the sample. The average age of the respondents is approximately 46.96 years, reflecting a middle-aged rural population. The level of education and political engagement is relatively low, with only 6.8% of respondents identified as communist party members. Most respondents are married, with a marriage rate of 81.6%, and the average health status is moderate to good, with a mean value of 3.609. In terms of household characteristics, 37.1% of households have property ownership. The average household size is 4.518 members, and the sanitary condition of households, measured on a scale from 1 to 10, has a mean value of 5.565, indicating moderate cleanliness. The villages'  social security conditions are relatively low, with an average score of 1.451. The mechanism variables within the study reveal several key insights. The average score for social capital, specifically in terms of neighbor's familiarity, stands at 3.828 with a standard deviation of 0.949, indicating that familiarity among neighbors is generally perceived to be moderate to high. Similarly, the trust that neighbors place in each other has an average score of 3.726 with a standard deviation of 0.806, suggesting a moderate to high level of trust within the community. When it comes to mutual support, the average score is slightly lower at 3.462 with a standard deviation of 0.977, which still places the level of support among neighbors above the midpoint of the scale. The rural community administration variable has an average value of 3.082 and a standard deviation of 3.442. The wide standard deviation suggests a varied implementation of grid management across different rural communities in the sample. Lastly, the village's collective economic income has an average value of 0.655 with a standard deviation of 0.475. This average value suggests that the collective economic income of the villages in the sample is moderate. These descriptive statistics suggest that the sample is representative of the rural population and aligns with the general characteristics of rural areas in the study region.

**Table 2:** Variable definitions and basic statistics.

Type	Variables	Definition	Mean	S.D.
**Dependent variable**	HWSW	1 = willing to participate in waste classification; 0 = unwilling	0.893	0.309
**Independent variable**	RLM	(number of people in household - number of people engaged in agricultural production for more than 3 months)/number of people in the household	0.556	0.245
**Control** **variable**	Gender	1 = male; 2 = female	0.524	0.499
	Age	The age of rural residents interviewed	46.595	14.416
	Communist party member	1 = communist party member; 0 = not communist party member	0.068	0.251
	Marriage	0 = not married; 1 = married	0.816	0.388
	Health	1 = very unhealthy; 2 = unhealthy; 3 = moderate 4 = healthy; 5 = very healthy	3.609	1.006
	Household property	1 = owner;0 = no	0.371	0.483
	Family member	The number of family members	4.518	2.067
	Household sanitary condition	From bad to good as represented by 1–10	0.565	0.496
	Villages’ social security conditions	1 = very bad; 2 = bad; 3 = moderate; 4 = good; 5 = very good	1.451	0.593
**Mechanism** **variable**	Social capital-Neighbor’s familiarity	1 = very little, 2 = relatively little, 3 = general, 4 = relatively much, 5 = very much	3.828	0.949
	Social capital-Neighbor’s trust	1 = very little, 2 = relatively little, 3 = general, 4 = relatively much, 5 = very much	3.726	0.806
	Social capital-Mutual supports	1 = very little, 2 = relatively little, 3 = general, 4 = relatively much, 5 = very much	3.462	0.977
	Rural community administration	1 = have grid management; 0 = None	3.082	3.442
	Village's collective economic income	The logarithm of the village's collective economic income	0.655	0.475

### Model setting

#### Baseline regression: Logit model.

We use the logit model for the baseline regression since the dependent variable in this paper (HWSW) is a discrete binary variable [51], which can be expressed as follows:


$${HWSW}_{i}^{*}=\alpha +\beta {RLM}_{i}+\gamma {Control}_{i}+\in$$ (1)

Where ${HWSW}_{i}^{*}$ is defined as the willingness of rural residents to engage in household waste classification. ${RLM}_{i}\;$indicates the ratio of non-farm employment, ${Control}_{i}$ represents a series of control variables as listed in Table 1, α, β, γ are the undetermined parameters, ∈ is the random disturbance term.

#### Correcting selection bias: GPS method.

This study might also consider that rural residents’ decisions regarding RLM may be self-selected rather than random. For example, Zhao and Jiang[52] pointed out that most migrated laborers are male and young, so families with more male and young laborers have a higher level of RLM. Therefore, this study uses the GPS method proposed by Hirano and Imbens [53] to correct this selection bias. The GPS approach is an alternative means of conducting propensity score-matching, which can remove self-select deviations in the comparison of treatment groups so that the effectiveness of RLM on HWSW can be assessed. In addition, the GPS approach does not need discrete continuous treatment so it can take advantage of rich information [54].

This study employs the following three-step approach to implement GPS [53].

(1) Estimating the $GPS(R_{i})$ of RLM under a given condition of restricting for an observed series of covariates

Considering that the RLM is a continuous variable, this paper uses the MLE method to estimate the conditional probability density of RLM. The equation is as follows:


$$g({RLM}_{i})|X_{i}\approx N\left\{f\left(\beta X_{i},\sigma^{2}\right)\right\}$$ (2)

Where $X_{i}$ represents the covariates, $f(\beta X_{i})$ represents the equation for all control variables including individual, household, and village characteristics. Due to the premise assumption of conditional independence that RLM is independent of HWSW, RLM with similar individual characteristics is randomly allocated. Based on the estimation of covariates, the $GPS(R_{i})$ of RLM is estimated as:


$${\hat{R}}_{i}=\frac{1}{\sqrt{2\pi {\hat{\sigma }}^{2}}}exp(-\frac{1}{\sqrt{2{\hat{\sigma }}^{2}}}{\left(g\left({RLM}_{i}\right)-\hat{\beta_{0}}-\hat{\beta_{1}}X_{i}\right)}^{2})$$ (3)

Where ${\hat{R}}_{i}$ represents the GPS of rural residents i. The estimate of Equation (3) can be obtained based on the calculation of Equation (2), and the results in Equation (3) are in preparation for the conditional expectation model.

(2) Estimating the conditional expectation of HWSW given GPS and RLM

The GPS method should also satisfy the balance test like the PSM method. After passing the balance test, we assessed the conditional expectation of HWSW as a function of two target variables—the RLM and the GPS variable. Thus, the average conditional expectation of HWSW can be calculated in Equation (4), and Equation (4) can also estimate whether the covariates in Equation (1) lead to any deviation.


$$E\left[Y_{i}|{RLM}_{i},R_{i}\right]=\alpha_{0}+\alpha_{1}{RLM}_{1}+\alpha_{2}{RLM}_{2}^{2}+\alpha_{3}{\hat{R}}_{i}+\alpha_{4}{\hat{R}}_{i}^{2}+\alpha_{5}{\hat{R}}_{i}*{RLM}_{i}$$ (4)

(3) Calculating the dose-response function and treatment-effect function

Through the above equations, we can predict the HWSW of each rural resident. Specifically, we use the processing variable (m) to replace the processing intensity value (RLM) and perform the score value (R) to the score value estimation function (r(m,X)). The average HWSW function DS(m) of each RLM value (m) can be calculated as the dose-response function below.


$$\hat{DS}(m)=\frac{1}{N}\sum_{i=1}^{N}\{{\hat{\alpha }}_{0}+{\hat{\alpha }}_{1}m_{i}+{\hat{\alpha }}_{2}m_{i}^{2}+{\hat{\alpha }}_{3}\hat{r}(m,x)+{\hat{\alpha }}_{4}{\hat{r}(m,x)}^{2}+{\hat{\alpha }}_{5}\hat{r}(m,x)e_{i}\}$$ (5)

Apart from the dose-response function, we also calculate the marginal effects of RLM based on the following treatment effect function.


$$\hat{TE}(m)=\hat{DS}\left(m_{i}+1\right)-\hat{DS}(m_{i})$$ (6)

#### Endogenous analysis: IV approach.

The above GPS method can only solve the endogenous problems that arise from selection bias caused by observable variables. However, it cannot solve the endogenous problems resulting from un-observable variables, reverse causality, and omitted variables. If there are endogenous problems of simultaneous causality and other un-observable factors influencing RLM and HWSW, the GPS approach's results are likely to be biased. Therefore, we use the IV method as a supplement to solve the endogenous problems caused by other factors. The 2SLS method is used in the IV method to deal with endogenous issues, which are set as follows:


$${RLM}_{i}=\mu +\varphi_{1}{Distance}_{i}+\varphi_{2}{Control}_{i}+\varepsilon_{i}$$ (7)

$${HWSW}_{i}=\tau +\omega_{1}{RLM}_{i}+\omega_{2}{Control}_{i}+\sigma_{i}$$ (8)

Where ${Distance}_{i}$ represents the IV which is the distance from the province to major ports, μ, τ, ${\;\phi }_{1}$, ${\;\phi }_{2}$, ${\;\omega }_{1}$, and $\omega_{2}$ are parameters to be estimated. $\varepsilon_{i}$, $\sigma_{i}$ are random perturbation terms. The remaining variables are the same as in Equation (1).

These two methods—the GPS approach and the IV method, allow us to address selectivity bias coming from both observable and un-observable characteristics and get an unbiased result of the impact of RLM on rural residents’ HWSW.

#### Mechanism analysis.

In order to test the mediating effect of mediate variables on the relationship between RLM and rural residents’ HWSW that the theoretical framework analyzed above, we refer to the literature of Li et al. [55], which introduces the interaction term between mediating variables and RLM (${RLM}_{i}\times M_{i}$) into function (1) and establish the following hybrid function (9):


$${HWSW}_{i}=\beta_{2}+\beta_{3}{RLM}_{i}+\beta_{4}{RLM}_{i}\times M_{i}+\beta_{5}M_{i}+\beta_{6}{Control}_{i}+\varepsilon_{i}+v_{i}+\delta_{i}$$ (9)

Where $M_{i}$ are mediation variables, social capital (neighbor's familiarity, neighbor's trust, and mutual support), rural community administration, and the village's collective economic income. The coefficient of the interaction term $\beta_{4}$ indicates whether mediation variables play a mechanism variables in the impact of RLM on rural residents’ HWSW.

## Empirical results

In this part, first and foremost, we investigate the impact of RLM on HWSW based on the Logit model. Then we use an empirical framework combining GPS and IV techniques to overcome the potential endogenous problems between RLM and HWSW. Furthermore, we run three robustness tests to improve the validity of our findings, and then we look into the heterogeneous effects of RLM on HWSW. Finally, we exhibit the mechanism of RLM’s effect on HWSW.

### Baseline results

Table 3 displays the benchmark findings grounded on the Logit model. Columns (1)-(2) present the estimated results without and with control variables, respectively. Column (3) reports the marginal effect of each variable. The results show that RLM significantly inhibits HWSW, which is in line with our expectations, and thus our **Hypothesis 1** is established. Specifically, the marginal effect results show that when RLM increases by 1%, the possibility of HWSW decreases by 3.5%. For example, the labor shortages induced by the COVID-19 pandemic have significantly disrupted waste management systems, leading to prolonged garbage accumulation in multiple regions and resulting in severe odor issues (please access: https://world.huanqiu.com/article/45JxmtJEm2v).

**Table 3:** Baseline results of the impact of RLM on HWSW.

Variable	Coefficient (1)	Coefficient (2)	Marginal effect (3)
**RLM**	-0.399^***^(0.147)	-0.350^*^(0.190)	-0.038^*^(0.020)
**Gender**		0.038(0.075)	0.004(0.008)
**Age**		-0.008^***^(0.003)	-0.001^***^(0.000)
**Communist party member**		0.782^***^(0.242)	0.084^***^(0.026)
**Marriage**		-0.066(0.050)	0.007(0.004)
**Health**		0.068^*^(0.037)	-0.019^*^(0.010)
**Household property**		-0.173^**^(0.076)	-0.018^**^(0.008)
**Family member**		-0.012(0.021)	-0.001(0.002)
**Household sanitary condition**		0.433^***^(0.085)	0.046^***^(0.009)
**Villages’ social security condition**		-0.225^***^(0.061)	-0.024^***^(0.007)
**Observations**	6,849	6,849	6849
**Pseudo R** ^**2**^	0.002	0.017	–

Notes: Standard errors are reported in parentheses. * p < 0.10, ** p < 0.05, *** p < 0.01, same as below tables.

For the control variable, the results mostly follow existing literature. Specifically, the coefficients of age, communist party member, health, house property, household sanitary condition, and villages’ social security condition are significant, indicating that these variables can affect HWSW. Age has a negative effect on HWSW, indicating that old rural residents’ HWSW is lower than that of young rural residents. This conforms with the findings of Wu et al.[56], who found that young people are more involved in waste classification because they have a higher need for their surroundings than older people. Being a communist party member can significantly promote peoples’ HWSW. This finding conforms with previous studies that communist party members have higher personal qualities and are therefore more willing to participate in environmental pollution management [57,58]. The healthier rural residents are, their HWSW is higher [10]. The possible explanation is that randomly stacked and unclassified domestic waste will produce a series of pollution and negative impacts, such as poor water quality, contaminated soil, and the spread of foul odors. It destroys the appearance and the living order in the village, generates germs, and causes diseases. House property ownership of a house is more likely to adopt HWSW [41]. Households with better sanitary conditions are more likely to adopt HWSW. The possible reason may be that people who live in better sanitary conditions are more conscious of their surroundings and are used to enjoying a better environment, and thus they have higher incentives to adopt HWSW [59]. Villages’ social security condition significantly promotes HWSW, which is consistent with the finding of Yang and Wang[60], who pointed out that improved villages’ security condition reduces the cost of governance, which in turn promotes HWSW.

### Correcting selection bias based on the GPS method

As we stated in the model setting section, there are three estimation steps when applying GPS. Thus, we present elaborate information on these three estimation steps.

#### First step estimation result: propensity scores calculation.

Table 4 displays the influencing factors of RLM. It shows that most of the selected covariates, such as age, marital status, health, number, and villages’ social security condition, have passed the significance test. The precise matching measurement is effective and reliable, which reflects the rationality of the covariates selection. Specifically, the coefficient of age is significantly negative at 1%, which reveals that younger rural residents are more likely to migrate. The possible reason may be that younger rural residents have higher socioeconomic incorporation than older rural residents, which is related to immigrants’ survival ability [61]. The coefficient of marital status is significantly positive at 5%. The possible reason might be that married individuals are more likely to seek higher-paying jobs to meet family needs, and are therefore more likely to choose migration [15]. The coefficient of health is significantly negative at 1%, implying that rural residents with better health are less likely to migrate. The possible reason may be that the better the health status of rural residents, the greater the possibility for laborers to work in agriculture, which makes them more willing to stay in rural areas [62]. The coefficient of family members is significantly positive at 5%. One possible reason might be that, in rural areas, agricultural production requires limited labor. To avoid resource waste and increase household income, families tend to transfer surplus labor to non-agricultural sectors, such as urban employment. The coefficient of villages’ social security conditions is significantly negative at 5%. The possible reason may be that a safe condition guarantees socialization and development for rural residents, which decreases their willingness to migrate [63].

**Table 4:** The influencing factors of RLM.

Variable	Coefficient	Robust Std. Err.
**Gender**	0.016	0.021
**Age**	-0.005^***^	0.001
**Communist party member**	0.019	0.049
**Marriage**	0.042^**^	0.015
**Health**	-0.022^**^	0.010
**Household property**	-0.025	0.021
**Family member**	0.341^***^	0.007
**Household sanitary condition**	0.022	0.026
**Villages’ social security condition**	-0.047^***^	0.017

#### Second step estimation result: balancing property and parameter estimation.

To test whether the control variable accorded with the balance requirements, RLM is divided into two groups based on the median value. We test whether there are significant differences between the means of each control variable between these two groups. The range of RLM for one group was 0 to 0.56 and for the other group was 0.56 to 1. Table 5 shows the results of the t-test for the difference in the means of the extent of RLM between the two sample groups with and without the use of GPS. The results show that the t-statistics for most of the control variables changed from significant to non-significant after adjustment with the GPS method, indicating that GPS effectively reduced the bias associated with differences in the control variables. The standard two-sided t-test passes the test of balance at a level below 0.01, implying that the requirements of the test of balance are met. In addition, the GPS method also verified that our covariates do not introduce deviation based on the parameter estimation (S1 Table).

**Table 5:** Balance test results.

Variables	Without using GPS adjustment		With using GPS adjustment	
[0-0.56]	[0.56-1]	[0-0.56]	[0.56-1]
**Gender**	4.039^***^	2.096^***^	0.531	-0.639
**Age**	-11.646^***^	-10.691^***^	-2.763^***^	2.763^***^
**Communist party member**	3.775^***^	7.820^***^	-0.049	-0.084
**Marriage**	-4.920^***^	-4.742^***^	-0.714	0.734
**Health**	2.495^**^	8.147^***^	1.152	-0.967
**Household property**	3.854^***^	7.197^***^	1.990^**^	-1.879^**^
**Family member**	-4.083^***^	-7.724^***^	0.824	-0.692
**Household sanitary condition**	4.657^***^	7.646^***^	2.157^**^	-2.074^**^
**Villages’ social security condition**	-8.276^***^	-10.257^***^	-0.037	-0.008

#### Third step estimation result: constructing the dose-response and treatment-effect curve.

**Fig 1:**
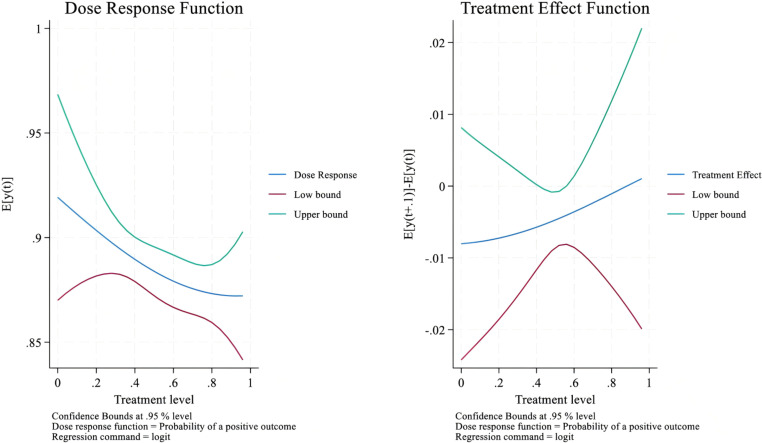
Average and marginal effects of RLM on HWSW.

The third step of estimation is to construct the dose-response and treatment-effect curve of RLM on HWSW based on the results of the above two steps. Specifically, we divide the value of RLM into five intervals from low to high and then estimate the causal effect of RLM on HWSW. Fig. 1 shows the average and marginal effects of RLM on HWSW, while the left graph represents the dose-response function curve, expressing the average effect of RLM on HWSW, and the right graph represents the treatment effect function curve, expressing the marginal effect of RLM on HWSW. The upper green and lower red curve represent the 95% confidence upper line and lower line of the GPS estimation function, respectively. The blue middle line between the upper green and lower red curves illustrates the causal effect function relationship between RLM and HWSW. From the trend of the blue middle line, it is obvious that it is a curve inclined to the lower right, indicating that the causal relationship between RLM and HWSW is negative in all five intervals of RLM. This result indicates that after the problem of sample selection bias is eliminated, RLM also negatively affects HWSW, which is consistent with our baseline results.

### Endogenous test based on IV approach

Although the above analysis concludes that RLM significantly inhibits HWSW, the GPS test results also support this conclusion. However, considering that there may be endogenous problems such as missing variables and mutual causality that may bias the estimation results, this study also solves the endogenous issues by using the IV approach. Following the methodology of Wu et al. [64], we use the distance from provinces to major ports as our instrumental variable (IV). The reason for selecting this IV is that the large-scale rural mobile labor force mainly flows toward coastal manufacturing and service industries due to the concentration of economic opportunities and infrastructure in these regions [65]. This implies that the distance to the coast significantly influences the transfer and mobility of rural labor. Proximity to ports facilitates access to non-agricultural employment opportunities, making it a strong determinant of rural labor migration. Furthermore, the distance from provincial capitals to ports does not logically have a direct relationship with HWSW. Waste separation behavior is primarily influenced by household-specific factors, such as education, health, and local policy initiatives, rather than geographic proximity to ports. This makes the distance to ports a suitable instrumental variable for addressing the endogeneity issue in our model.

Table 6 presents the results of the endogeneity test. Column (1) of the first stage shows that the distance from provinces to major ports significantly promotes RLM at the 5% significance level, which satisfies the relevance condition for a valid instrumental variable (IV). In addition, the F-statistic exceeds the critical value of 10, indicating that the IV is not weak. Column (2) of the second stage shows that RLM significantly negatively impacts HWSW at the 10% significance level. The estimated coefficient (-0.717) is larger in absolute value than the coefficient in the baseline regression (-0.350), suggesting that the inhibitory effect of RLM on HWSW becomes more pronounced after addressing the endogeneity issues caused by simultaneous causality and omitted un-observable variables.

**Table 6:** Endogenous test results.

Variable	2SLS	
First Stage RLM	Second Stage HWSW
(1)	(2)
**Distance to ports**	0.015^**^(0.007)	
**RLM**		-0.717^*^(0.410)
**Control variables**	YES	YES
**F value**	401	
**Wald value**		64.66
**Observations**	6,849	6,849

Note: The control variables are consistent with Table 3.

### Robustness test

To improve the reliability of our results, we conduct three robustness tests including changing the definition of HWSW, changing the regression method, and winsorizing extreme values.

(1) Changing the definition of HWSW. According to previous literature, in some areas, HWSW might be supervised by the village, which may involve the establishment of designated drop-off points where specialists assist with waste sorting[15]. To account for this, we use the willingness to take household waste to a designated drop-off point as an alternative measure of HWSW. This is based on the following question in the CLDS survey: “Is your household willing to take your household waste to a designated drop-off point?” Responses range from 0 (no) to 1 (yes). The results are presented in Columns (2)-(3) of Table 7. Our findings are largely consistent with the main results, with similar directions and levels of statistical significance, supporting the robustness of our conclusions.
(2) Changing the regression method. In addition to using the Logit model for regression, we also employ machine learning methods and the probit model to verify the robustness of the baseline results. Machine learning methods are particularly advantageous as they can capture potential non-linear relationships and do not rely on strict distributional assumptions, making them a flexible tool for robustness checks [66]. Meanwhile, the Probit model, which assumes a standard normal distribution for the error term, provides an alternative approach to modeling binary dependent variables and allows us to test the sensitivity of the results to different distributional assumptions [67]. The results, displayed in Columns (2)-(3) of Table 7, are broadly consistent with the main findings in terms of the direction and statistical significance of the coefficients, further supporting the reliability of our conclusions.
(3) Winsorizing extreme values. To ensure that the effect of RLM on HWSW is not affected by extreme values, we delete the up and bottom 1% of the sample for another robustness test. The results in Column (4) of Table 7 further prove the robustness of the previous conclusion because the results are consistent with the baseline model after eliminating the outliers.

**Table 7:** Robustness test results.

Variable	Changing the definition of HWSW	Changing the regression method		Winsorzing extreme values
		Machine learning method	Using the Probit method	
(1)	(2)	(3)	(4)
**RLM**	-0.003^***^(0.001)	-0.039^*^ (0.023)	-0.179^*^(0.096)	-0.363^*^(0.196)
**Control variables**	YES	YES	YES	YES
**Observations**	6,849	6,849	6849	6,849
**R-squared**	0.028	–	0.027	0.017

Note: The control variables are consistent with Table 3.

### Heterogeneity analysis

As stated in the baseline regression results, RLM significantly restrained HWSW, but the above conclusion does not consider its inherent differences. In this section, we further examine the heterogeneity effect of RLM on HWCW in rural residents of different regions, education, and income levels.

For the test of regional heterogeneity, we divide the sample into Eastern (Z = 0), and Midwestern (Z = 1). The economic development, cultural backgrounds, infrastructure construction, and rural residents’ living habits varied a lot in Eastern and Midwestern China and thus has large regional heterogeneity in different regions [68]. Column (1) of Table 8 presents the heterogeneous analysis by region. The results suggest that, compared with the Eastern region, the negative effect of RLM on HWSW is more pronounced in the Midwest region. Specifically, while the main effect of RLM on HWSW is -0.586 and statistically significant, the coefficient of the interaction term is -0.361, which is statistically significant at the 10% level. This indicates that the effect of RLM on HWSW varies significantly between the two regions. A possible explanation is that the lower level of economic development in Midwestern regions limits the resources available to rural households, such as time and money [69]. When labor migrates, households may prioritize basic livelihood needs over waste management. This resource constraint is more severe in these regions, which amplifies the negative impact of labor migration on waste management behavior. In contrast, households in eastern regions, with higher income levels, may have more resources to manage waste. They can rely on hired labor or community services, which reduces the negative impact of labor migration.

**Table 8:** Heterogeneity analysis results.

Variable	HWSW		
(1)	(2)	(3)
**RLM**	-0.586^*^(0.334)	-0.367^*^(0.198)	-2.033^*^(1.155)
**RLM** ×R **egion**	-0.361^*^(0.217)		
**RLM** × **Education**		-0.348^*^(0.189)	
**RLM** × **Income**			-0.254^*^(0.135)
**R-squared**	0.016	0.016	0.021
**Observations**	6,849	6,849	6849

With regard to education, it is widely recognized as a key factor influencing environmental behavior and waste sorting behavior with significant differences in participation across regions due to varying education levels [70]. Therefore, we segmented the sample based on the median of rural residents’ education into two sub-samples: the lower-educated group (Z = 1) and the higher-educated group (Z = 0). Columns (2) of Table 8 presents the results of the heterogeneous analysis based on rural residents’ education levels. The findings suggest that the effect of RLM on HWSW is more pronounced for rural residents with lower education levels. A possible explanation might be that the weaker environmental awareness among less-educated rural residents stems from their limited exposure to environmental education and awareness campaigns, which are often more accessible to rural residents with higher educational attainment. This is evident in real-world examples. For instance, the recycling rate is less than 10% in countries like Iran and Turkey, where lower levels of education are prevalent [71,72]. Furthermore, rural residents’ focus on immediate economic survival—often a result of RLM—may divert attention from long-term environmental issues, further diminishing their motivation to participate in HWSW [73].

As for the households’ income, these differences in attitudes and capabilities towards waste separation are primarily due to varying income levels among households [10]. Therefore, we segmented the sample based on the median of households’ income into two sub-samples: the lower-income group (Z = 1) and the higher-income group (Z = 0). Columns (3) of Table 8 present results by households’ income. It suggests a larger RLM effect on HWSW from lower income. A possible reason is that low-income families are often more reliant on household labor to complete daily tasks and agricultural activities, including waste sorting and disposal [74]. When family members migrate to work, the household's labor resources are significantly reduced. This labor shortage decreases the time and energy available for HWSW. Such dependency is particularly pronounced in low-income households, as they typically lack external resources, such as hired labor or paid waste management services, to compensate for the loss.

### Mechanism analysis

The previous empirical results have unveiled that RLM can significantly negatively affect HWSW. In this section, we turn to investigating the potential mechanisms of RLM on HWSW. According to the analytical framework in Section 2, social capital, rural community administration, and the village's collective economic income are the possible mechanisms.

Table 9 shows the results of the mechanism analysis. Columns (1)-(3) report the social capital mechanism results. Column (1) introduces the interaction term between RLM and neighbor's familiarity (RLM × neighbor's familiarity) to capture the mechanism effect of neighbor's familiarity. The coefficient of the interaction term is negative and significant at the 1% level, this indicates that households with higher levels of neighbor's familiarity experience a greater negative impact from RLM on their HWSW compared to those with lower levels of neighbor's familiarity, thus validating Hypothesis 2. Similar patterns are observed for the neighbor's trust and neighbor support degrees. These results suggest that reductions in social capital (as a result of RLM) weaken rural residents’ HWSW. In response, local governments should establish community centers or hubs to strengthen social connections and promote environmental education. These centers can host workshops on waste sorting and environmental protection. Existing public facilities, such as village schools or town halls, can be repurposed to minimize costs. Funding can come from local budgets or public-private partnerships. In addition, digital platforms or mobile applications can be developed to facilitate communication among residents, share information on environmental initiatives, and organize clean-up activities.

**Table 9:** Mechanism analysis results.

Variable	Subjective Norms				Perceived behavioral control
Neighbor’s Familiarity	Neighbor’s Trust	Mutual Support	Rural community administration	Village's Collective economic income
(1)	(2)	(3)	(4)	(5)
**RLM**	-0.329^*^ (0.182)	-0.316^*^ (0.182)	-0.325^*^ (0.182)	-0.446^*^ (0.246)	-0.331^*^ (0.182)
**RLM** × **Neighbor’s Familiarity**	-0.125^***^ (0.044)				
**RLM** × **Neighbor’s Trust**		-0.168^***^ (0.047)			
**RLM** × **Mutual Support**			-0.075^*^ (0.040)		
**RLM** × **Rural community administration**				-0.302^***^ (0.075)	
**RLM** × **Village's Collective economic income**					-0.022^**^ (0.011)
**R-squared**	0.061	0.017	0.019	0.019	0.016
**Observations**	6,849	6,849	6849	6,849	6,849

Note: The control variables are consistent with Table 3.

Column (4) introduces the interaction term between RLM and rural community administration (RLM × rural community administration) to capture the mechanism effect of village management. The coefficient of the interaction term is negative and significant at the 1% level, thus validating Hypothesis 2. This indicates that villages with higher levels of village management experience a greater negative impact from RLM on HWSW compared to those with lower levels of village management. A potential explanation is that villages with stronger administrative structures may rely more heavily on collective participation, which is disrupted by the out-migration of key labor resources. To mitigate the weakening of village administration caused by labor migration, capacity building for local leaders is essential. Local governments can organize training programs that focus on governance, resource allocation, and community engagement. For example, workshops could equip village leaders with the skills to design waste management strategies and allocate resources more efficiently, even when facing labor shortages due to migration. Strengthening the leadership capacity at the village level can help ensure the continuity and effectiveness of waste management initiatives.

Column (5) introduces the interaction term between RLM and the village's collective economic income (RLM × village's collective economic income) to capture the mechanism effect of the village's collective economic income. The coefficient of the interaction term is negative and significant at the 5% level, thus validating Hypothesis 2. This suggests that villages with higher levels of the village's collective economic income experience a greater negative impact from RLM on HWSW compared to those with lower levels of village's collective economic income. One possible explanation is that villages with higher village's collective economic income may have more established waste management systems or practices that are disrupted by RLM. Strengthening a village's collective economic income can help mitigate these disruptions by providing the financial resources needed to adapt waste management systems to changing labor dynamics. For instance, promoting the establishment of biogas plants that convert organic waste into energy can reduce waste emissions while generating economic benefits for the village. Such initiatives not only alleviate the economic pressure caused by labor migration but also support sustainable waste management practices.

## Conclusion and policy recommendations

Promoting rural residents’ HWSW is the most critical way to realize household waste management in rural China. However, few studies have been dedicated to examining China’s massive RLM on HWSW. Based on a nationwide sample including 6849 rural residents, this paper provides the first evidence of the relationship between RLM and HWSW. Our results indicate that: first, RLM significantly inhibits HWSW. A series of robust probes validate that this finding is robust and credible. Second, heterogeneity analysis reveals that the negative impact of RLM on HWSW is greater for people who live in Midwest regions, with low education and income level. Third, according to a mechanism analysis, social capital, rural community administration, and the village's collective economic income are the main mechanisms by which RLM has a negative impact on HWSW.

According to the above research conclusions and the current development situation of HWSW in rural China, we propose the following recommendations.

First, policies should focus on creating attractive opportunities for skilled and semi-skilled workers to return to their hometowns, particularly in regions where waste management systems are underdeveloped. For example, local governments could incentivize return migration by providing subsidies or grants for individuals who initiate or lead community-based environmental projects, such as waste recycling, composting, or biogas production. These initiatives not only address local waste management challenges but also create employment opportunities for returning workers. In addition, fostering the development of local industries with high potential for job creation and economic growth is essential to attract talent back to rural areas. For instance, regions with abundant natural or cultural resources could prioritize the development of eco-tourism projects. Local governments might offer financial support for the establishment of eco-friendly accommodations, the restoration of cultural heritage sites, or training programs for returning workers to become tour guides or eco-tourism entrepreneurs. Similarly, in areas with strong agricultural bases, efforts could focus on developing agricultural product processing, branding, and e-commerce platforms to connect rural producers with broader markets. These initiatives could provide skilled workers with opportunities to apply their expertise in marketing, logistics, or technology, making rural areas more appealing for return migration. By aligning these efforts with the specific resource endowments and economic conditions of each region, the government can create a favorable environment that not only attracts talent back to rural areas but also fosters sustainable development and environmental management.

Second, strengthening investments in waste management infrastructure is crucial, particularly in economically disadvantaged regions. Priority should be given to establishing recycling centers, composting facilities, and waste collection systems in areas where such infrastructure is lacking. Local governments should also allocate resources to maintain and upgrade existing facilities to ensure their long-term functionality and effectiveness. To align infrastructure development with local employment creation, governments can design waste management projects that actively involve community members in the construction, operation, and maintenance processes. This approach not only improves waste management outcomes but also generates employment opportunities. For instance, local cooperatives or small enterprises could be supported to manage recycling centers or composting facilities, fostering community ownership and participation. Additionally, integrating waste management infrastructure with community-based employment programs can simultaneously enhance environmental outcomes and provide sustainable livelihood opportunities for rural residents.

Despite the findings above, this study has potential limitations and analytical challenges concerning research data. Specifically, the omission of certain variables related to waste management infrastructure, such as the availability of garbage treatment facilities and the number of garbage bins near households, poses a limitation. These factors may play a crucial role in shaping HWSW, but due to data constraints and multicollinearity concerns, they were not included in the final analysis. Additionally, the study relies on cross-sectional data, which limits the ability to capture dynamic changes in household behavior over time. The lack of longitudinal data restricts the exploration of causal relationships and the long-term effects of various influencing factors on HWSW. Future studies could address these limitations by collecting more granular and comprehensive data on waste management infrastructure and other contextual factors. Furthermore, utilizing longitudinal panel data would enable a deeper understanding of the temporal dynamics and causal mechanisms underlying HWSW.

## Supporting information

S1 Table The second step of the GPS method. (DOCX)
