# Supplementary material for: Rural labor migration and household waste separation willingness: Evidence from 6849 rural residents in China
Source: PLoS One. 2025 May 28;20(5):e0321459. doi: 10.1371/journal.pone.0321459 (PMC12118849; doi:10.1371/journal.pone.0321459)
Supplement: S1 Table — (DOCX) [file pone.0321459.s001.docx]

The second step of the GPS method is to apply the results obtained in the first step to calculate the conditional expected value of rural residents’ HWSW. Considering that the treatment variable in this chapter, labor transfer, is a continuous variable, this section calculates the average conditional expected value of rural residents’ HWSW as a function of labor transfer and the propensity score matching value. Specifically, this section uses rural residents’ HWSW as the outcome variable and employs labor transfer and the propensity score matching value obtained in the first step as explanatory variables for OLS estimation. The results are presented in Table S1. The results show that none of the variables are significant.

**S1 Table. The second step of the GPS method.**

| **Variables** | HWSW | |
| --- | --- | --- |
|  | **Coefficient** | **Robust Std. Err.** |
| **RLM** | -3.072 | 8.493 |
| **RLM^2^** | -0.395 | 1.900 |
| **R** | -5.290 | 30.161 |
| **R^2^** | 8.370 | 24.463 |
| **RLM*R** | 4.970 | 13.351 |
| **常数** | 3.417 | 10.097 |
